# Insights into the shape-dependent effects of polyethylene microplastics on interactions with organisms, environmental aging, and adsorption properties

**DOI:** 10.1038/s41598-023-49175-1

**Published:** 2023-12-13

**Authors:** Ula Rozman, Barbara Klun, Aleksandra Kuljanin, Tina Skalar, Gabriela Kalčíková

**Affiliations:** Faculty of Chemistry and Chemical Technology, University of Ljubljana, 113 Večna pot, 1000 Ljubljana, Slovenia

**Keywords:** Environmental sciences, Environmental chemistry, Environmental impact

## Abstract

The shape-dependent effects of microplastics have been studied in the context of ingestion but have not been considered in other environmental processes. Therefore, we investigated how the shape of polyethylene microplastics (spheres, fragments, and films) affects interactions with plants, aging, and their adsorption properties. The shape had no effect on the growth rate and chlorophyll content of duckweed *Lemna minor*, but the fragments strongly adhered to the plant biomass and reduced the root length. The adsorption process of the model organic compound (methylene blue dye) was described by the same kinetic model for all shapes—the experimental data best fit the pseudo-second order model. However, twice as much methylene blue was adsorbed on films as on fragments and spheres. During environmental aging, most biofilm developed on films. The biofilm on spheres contained significantly less photosynthetic microorganisms, but twice as much extracellular polymeric substances (EPS) as on fragments and films. This suggests that the attachment of microorganisms to spherical particles is limited and therefore more intensive production of EPS is required for stable biofilm formation. From the results of this study, it is evident that the shape of microplastics significantly affects not only ecotoxicity but also other environmentally relevant processes.

## Introduction

Recently, microplastics (MPs), solid, insoluble plastic particles ranging in size from 1 to 1000 µm^1^, have attracted particular interest due to their abundance in the environment and potential impact on biota and human health^2^. MPs, however, are not a uniform compound or material, but they are characterized by extreme variability, as they can be made from any polymer ever produced, contain different additives in the polymer matrix, and have different shapes and sizes^3,4^.

The polymer type and the presence of additives play an important role in evaluating the ecotoxicity of MPs, as MPs composed of pure polymer often do not have negative effects, whereas those with additives or with unreacted monomers do^5–7^. Polymer type also affects MPs interactions with other contaminants, as certain polymer types adsorb contaminants to a greater extent^8^. Another important characteristic of MPs is their size, and therefore size-dependent ecotoxicological effects have been widely studied^9,10^. Smaller particles can be ingested by organisms and distributed in the circulatory system and organs^11,12^, while larger particles are simply excreted^13^. Small MPs have been shown to be more mobile in sediments, so size can also affect their fate in the environment^14^.

In addition, MPs exist in various shapes, such as spheres, fragments, films, and fibers, however shape-dependent interactions and ecotoxicological effects are not well known. Some researchers have focused on the shape-dependent ingestion of MPs, their retention in the digestive tract, and the resulting ecotoxicological effects^13,15–19^. In addition, Zhang and Choi^20^ investigated a shape-dependent drag model for settling velocity of MPs. However, interactions between MPs of different shapes and biota beyond ingestion, e.g., via bioadhesion^21^, are not known. It is also not clear whether the shape of MPs can influence aging in the environment (e.g., development of biofilm = biofouling) or interaction with contaminants in their surrounding environment. However, these aspects are crucial for understanding the ecotoxicological effects and environmental behavior of different MPs^22,23^.

In this context, the aim of this study was to investigate how polyethylene (PE) MPs of a similar size but with three different shapes, namely spheres, fragments, and films differ in terms of (i) plant-MPs interactions (ecotoxicity and adhesion of MPs to biomass), (ii) adsorption capacity, and (iii) biofouling (focusing on changes in biofilm development and composition). The main motivation for this study is that these aspects have never been investigated before for different MP shapes. By covering the three major shapes of MPs for one polymer, we will be able to link potential impacts directly to the shape, which will greatly contribute to a better understanding of MPs fate and their ecological implications in the aquatic environment.

## Results

### Characterization of microplastics

**Figure 1:**
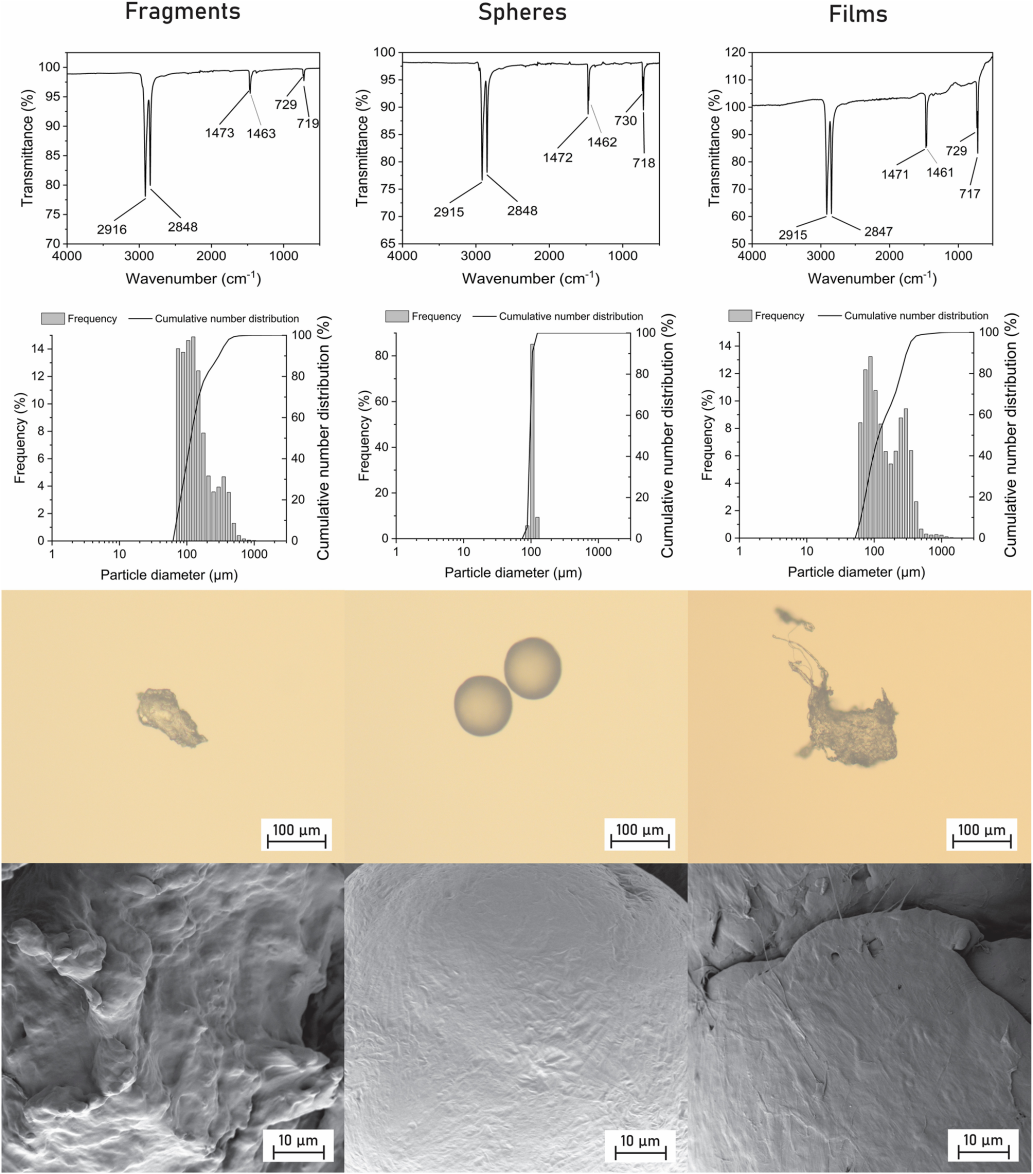
FTIR spectra (1st row), number particle size distributions (2nd row), images from optical microscope at ×100  magnification (3rd row), and FE-SEM images at ×4000  magnification (4th row) of fragments, spheres, and films.

MPs used in this study had different shape, but the same chemical composition (Fig. 1). Based on the FTIR analysis, it was confirmed that all three types of MPs were made of polyethylene (PE), with characteristic peaks at 2915 cm^−1^ (C–H asymmetric stretching), 2848 cm^−1^ (C–H symmetric stretching), 1463 cm^−1^ (CH_2_ bending), and 719 cm^−1^ (CH_2_ rocking) ^24^. Spheres were monodispersed, while fragments and films were polydispersed due to the way they were prepared (i.e., by chopping and grinding) and therefore the particle size was more widely distributed. However, the majority of MPs of each type were around 100 µm. The fragments had an irregular shape and irregular surface, while the spheres had a uniform shape with smooth surface. The films were thin and had irregularities on the surface and exhibited tendency to fold. The number of particles per mass was comparable for fragments and films, but much higher for spheres (Table 1). The specific surface area was low and comparable for all three types of MPs. The degree of crystallinity was the highest in spheres > films > fragments and the same trend was seen also for crystallite size (Table 1).

**Table 1:** Characteristics of tested microplastics.

	Fragments	Spheres	Films
Mean number particle size (µm) (mean ± SD, n = 3)	149 ± 75	97 ± 6	161 ± 99
Number of particles per mass (particles/mg)	96	2708	65
Specific surface area (m^2^/g)	0.0178	0.0216	0.0144
Degree of crystallinity (%)	39.2	98.8	53.7
Crystallite size (nm)	9.0	30.5	18.3

### Interactions with plants

**Figure 2:**
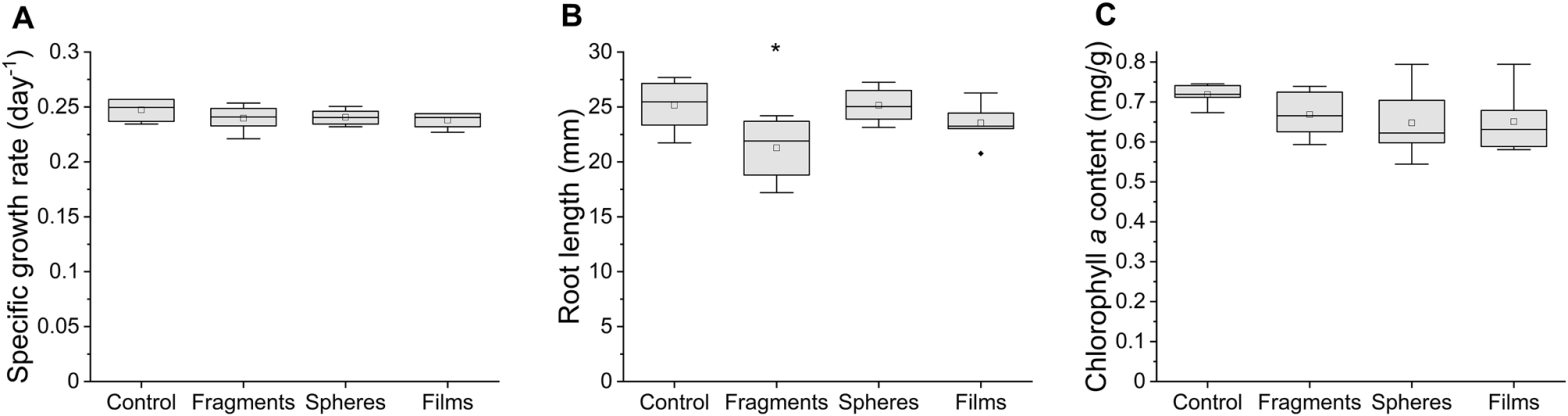
The effects of fragments, spheres, and films on specific growth rate (A), root length (B), and chlorophyll *a* content (C) of *Lemna minor* (n = 6). *Significant difference compared to control (p < 0.05). Line—median, square—mean, box—25‒75%, whiskers—range within 1.5IQR, deltoid—outlier.

The ecotoxic effects of fragments, spheres, and films on *Lemna minor* were investigated, namely the effects on the specific growth rate, root length, and chlorophyll *a* content (Fig. 2). The results showed that MPs did not significantly affect the specific growth rate, as the reduction was 7 ± 3% (*p* = 0.379), 3 ± 2% (*p* = 0.567), and 4 ± 3% (*p* = 0.280) for fragments, spheres, and films, respectively, compared to control (Fig. 2A). Chlorophyll *a* content was also not significantly affected by any shape of MPs (inhibitions caused by fragments, spheres, and films were 13 ± 6% (*p* = 0.531), 10 ± 12% (*p* = 0.268), and 9 ± 11% (*p* = 0.306), respectively) (Fig. 2C). The only significant negative effect was observed as a reduction in the root length by fragments (19 ± 10%, *p* = 0.021), while the inhibition on root length by spheres and films was not significantly different from the control (4 ± 4% (*p* = 1.000) and 9 ± 7% (*p* = 0.652), respectively) (Fig. 2B).

The shape of MPs had a significant effect on both, the number of adhered MPs and on the binding strength of MPs to plant biomass (Table 2). Fragments adhered to the highest extent and also the number of strongly and weakly adhered fragments per plant mass was the highest compared to adhered spheres and films. The total number of adhered spheres was higher than the total number of adhered films, but most spheres were weakly adhered. Statistical testing showed that the number of weakly adhered fragments and spheres was comparable and not statistically significant (p = 0.642). Furthermore, lower number of films were weakly adhered to plant biomass compared to fragments and spheres, but the difference was not statistically significant (p = 0.179 and 0.595, respectively). On the other hand, the difference in the number of strongly adhered MPs was statistically significant between all types of MPs (p < 0.001, p = 0.003, p = 0.020 for comparisons between fragments and spheres, fragments and films, and films and spheres, respectively). The shape of MPs therefore had a significant effect only on the number of strongly adhered particles.

**Table 2:** Strong and weak adhesion of fragments, spheres, and films (mean ± SD, n = 4) and number percentage of total adhered microplastics to the biomass of *Lemna minor.*

Shape	Weak adhesion (particles/mg)	Strong adhesion (particles/mg)	Total adhered microplastics (%)
Fragments	2.23 ± 1.20	0.32 ± 0.08	42
Spheres	1.68 ± 1.08	0.02 ± 0.03	30
Films	1.10 ± 0.25	0.15 ± 0.07	21

### Adsorption properties

**Figure 3:**
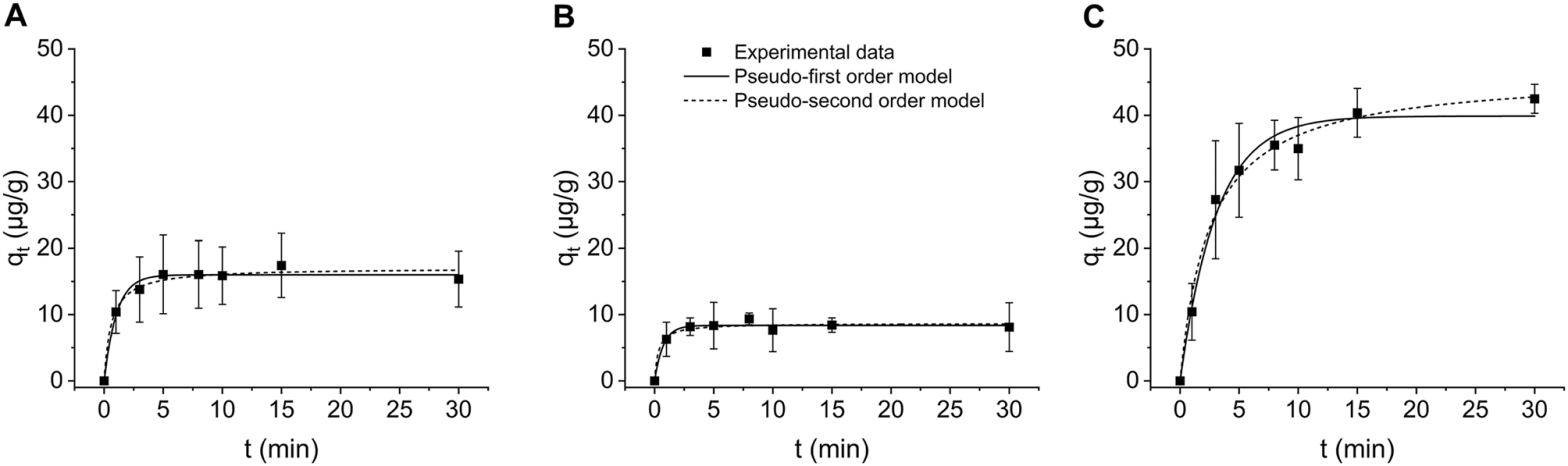
Non-linear kinetic fits of experimental data (n = 4) to pseudo-first order and pseudo-second order for adsorption of methylene blue dye to (**A**) fragments, (**B**) spheres, and (**C**) films.

Adsorption of the methylene blue dye (MB) occurred at different rates when adsorbed on fragments, spheres, and films, with maximum adsorption capacity reached after 15, 8, and 30 min, respectively (Fig. 3). Adsorption kinetic was determined by applying two kinetic models to the experimental data, namely the pseudo-first and pseudo-second kinetic models. Both models showed relatively good fit with the experimental data, as *R*^2^ was always above 0.900 (Table 3). However, the experimentally determined concentration of adsorbed MB at the equilibrium (*q*_*e,exp*_) was similar to the calculated value (*q*_*e,cal*_) from the pseudo-second order model for all three types of MPs (Table 3), indicating better agreement between the experimental data and the pseudo-second order model.

**Table 3:** Parameters for pseudo-first and pseudo-second kinetic models for methylene blue dye (MB) adsorption on fragments, spheres, and films.

Shape	*q*_*e,exp*_ (µg/g)	Pseudo-first-order model			Pseudo-second-order model		
		*K*_*1*_ (1/min)	*R*^2^ (–)	*q*_*e,cal*_ (µg/g)	*K*_2_ (g/(µg·min))	*R*^2^ (–)	*q*_*e,cal*_ (µg/g)
Fragments	17.4 ± 4.8	0.95	0.981	15.99	0.096	0.983	17.04
Spheres	9.4 ± 0.9	1.38	0.975	8.36	0.354	0.967	8.67
Films	42.5 ± 2.2	0.32	0.983	39.88	0.008	0.989	46.38

*q*_*e,exp*_ experimental MB concentration at equilibrium, *q*_*e,cal*_ calculated MB concentration at equilibrium, *R*^*2*^ correlation coefficient, *K*_*1*_ pseudo-first order rate constant, *K*_*2*_ pseudo-second order rate constant.

When reaching the maximal adsorption capacity, 17 ± 5%, 9 ± 1%, and 43 ± 2% of the MB was adsorbed on fragments, spheres, and films, respectively, and the statistical testing showed that there were significant differences in the maximum amount of MB adsorbed between films and spheres/fragments (both *p* < 0.001), but not between spheres and fragments (*p* = 0.056).

### Biofouling

The amount of biofilm developed on the surface of MPs was highest on films, followed by fragments and spheres. No statistically significant difference between the amount of biofilm formed on spheres and fragments (*p* = 0.175) was observed, while the amount of biofilm formed on the films was significantly higher compared to the amount of biofilm formed on the spheres (*p* = 0.007) and fragments (*p* = 0.035) (Table 4).

**Table 4:** Characteristics of the biofilm formed on the surface of fragments, spheres, and films (mean ± SD, n = 3).

Shape	The amount of biofilm on MPs (%)	EPS (mg_glucose_/g_biofilm_)	Chlorophyll *a* (mg/g_biofilm_)
Fragments	36 ± 6	2.67 ± 0.37	0.90 ± 0.11
Spheres	21 ± 1	4.05 ± 0.69	0.31 ± 0.12
Films	58 ± 10	1.99 ± 0.14	1.05 ± 0.15

*EPS* extracellular polymeric substances.

Furthermore, the biofilm developed on the spheres contained significantly more EPS in comparison with biofilm developed on the fragments (*p* = 0.028) and films (*p* = 0.005), while the concentration of EPS was similar for biofilm on fragments and films (*p* = 0.187) (Table 4).

The amount of chlorophyll *a* in biofilm also significantly differed between different shapes of MPs (Table 4). Biofilm on the spheres contained significantly lower amount of chlorophyll *a* compared to fragments (*p* = 0.010) and films (*p* = 0.004), while no statistically significant difference of the chlorophyll *a* concentration was observed between fragments and films (*p* = 0.415).

## Discussion

MPs are a very diverse group of pollutants, their variability is in fact infinite, as they are made of different materials, vary in size^3,25^, change their properties during their lifetime in the environment^26^, and interact with different pollutants^27^. They can also have different shapes and have been found in the environment as fragments, films, pellets, fibres, and foams, while MPs with ideal spherical shapes are not present in the environment but are still the most commonly used in MP research^3^. Therefore, we aimed to investigate whether the shape of MPs plays an important role in the interactions of MPs in the aquatic environment.

The MPs used in this study were all made of the same polymer—polyethylene—and were produced in comparable sizes but they differed in shape (Fig. 1). However, their shape can also determine their properties such as surface morphology and degree of crystallinity. For example, fragments were made by grinding larger pieces of PE^28^ and their surface morphology was more complex than that of spheres made by polymerization in the chosen shape and therefore have a smooth, uniform surface. The same is true for the degree of crystallinity. For some plastic products, the degree of crystallinity is lower because the amorphous regions are responsible for their flexibility^29^. Also, the more crystalline the material is, the longer is the chain order, which results in a larger crystallite size^30^. Therefore, MPs that differ in shape will always differ in some of their properties (e.g., morphology, crystallinity), although they are made of the same material.

The shape of MPs has been mainly linked to ecotoxicological effects when MPs are ingested by animals^13,15–19^, however, as shown here, the shape is also an important factor when considering other interactions with aquatic organisms such as bioadhesion (Table 2). In fact, fragments were the only shape of MPs that had an effect on the roots of aquatic plants (Fig. 2). This is most likely related to bioadhesion (Table 2), as they adhered to the highest extent to plant biomass compared to spheres and films. Although most fragments were weakly adhered, the proportion of strongly adhered MPs was also the highest for fragments (Table 2). They had irregularities on their surface that can better hook on the plant surface compared to MPs with smooth surface and the resulting negative effect was most likely due to their sharpness (Fig. 1), which can compromise root cell membrane integrity^31^. On the other hand, the films also had an irregular shape and surface (Fig. 1), but the fold material seems to be more flexible and therefore did not affect the roots. Similarly, irregularly shaped natural particles from wood did not affect roots of duckweed because of the softness of wood^5,22^. The spheres had smooth surface with an absent abrasive effect and they adhered to plant biomass to a lesser extent and only via weak interactions which is in an agreement with results of Mateos-Cárdenas et al.^32^ who observed no effects on the root length of duckweed when exposed to high concentrations of PE spheres (10–45 µm).

The adsorption process of all tested MPs was described by the pseudo-second order model which indicates that the rate controlling step in the adsorption process is the binding of the adsorbate to the adsorbent^27,33^. Comparison of the pseudo-second order rate constants (*K*_*2*_, Table 3) showed that the adsorption process was the fastest for the spheres, followed by the fragments and the films, which was consistent with the time needed to reach the maximum adsorption capacity (spheres > fragments > films). On the other hand, the adsorption capacity significantly differed for different shapes of MPs. Almost twice as much dye was adsorbed on the films as on the fragments and spheres (Fig. 3). This is unexpected as all shapes of MPs had comparable specific surface area (Table 1). In addition, both fragments and films were irregularly shaped (Fig. 1), had similar particles size distribution, comparable degree of crystallinity (Table 1), and therefore, the adsorption capacity should be comparable^34^. In addition, the fragments and spheres had completely different shape and surface morphology (Fig. 1), but the amount of the adsorbed dye was comparable. Similar results were obtained by Almeida et al.^35^, who reported a significantly higher amount of copper adsorbed on PE films compared with PE spheres and PE fragments (size unknown). In contrast, Yu et al.^36^ reported fivefold higher adsorption capacity of PE fragments for adsorption of tetrabromobisphenol a compared to PE spheres of a similar size. It is evident that the adsorption capacity did not depend only on the specific surface area, surface morphology, or degree of crystallinity, as this has already been documented, but that the shape itself (or other parameters) may also play a role in the adsorption process, which requires further investigation of the contribution of MPs properties to the adsorption process.

During biofouling, the amount of biofilm developed on the surface was comparable between fragments and films in terms of all parameters investigated i.e., amount of biofilm, EPS, and chlorophyll *a* content (Table 4). On the other hand, the amount of biofilm was the lowest on spheres, but there were about twice more EPS compared to fragments and films (Table 4). The EPS secreted by microorganisms plays an important role in the attachment to the surface and to ensure the stability of the biofilm^37,38^. Therefore, higher amount of EPS in biofilm developed on spheres indicated that microorganisms had to secrete significantly more EPS in order to adhere to the smooth surface of spheres. The content of chlorophyll *a* was the lowest on spheres suggesting that the smooth surfaces are not favourable for attachment of photosynthetic microorganisms such as algae and cyanobacteria that are able to attach to PE fragments^39^. Overall, it seems that the differences in the biofouling can be caused by the surface morphology as microorganisms can easily attach to heterogeneous surfaces^40^ and do not have to produce as much EPS as for attachment on smooth surface of spherical particles. This is in agreement with results of Parrish and Fahrenfeld^41^ who investigated biofilm formation on PE fragments, PS spheres, and glass spheres and suggested that the biofilm development is more dependent on the morphology than chemical composition, as glass and PS spheres had similar number of cells on the surface, while the number of cells on PE fragments was higher. Similarly, Cheng et al.^42^ reported significantly higher bacterial heterotrophic activity of biofilm on PE fragments in comparison with biofilm on PE spheres and authors suggested that the difference in the activity occurred due to different packing of bacteria in biofilm and consequently different biofilm structure. However, we argue here, that also the degree of crystallinity or contra—the presence of amorphous regions can be behind the increase biofouling on fragments and films as these regions in polymer are tent to be first utilized by attached microorganisms^43,44^. This is, thus, most likely the reason why limited amount of biofilm was developed on spheres which had nearly 100% degree of crystallinity (Table 1).

**Figure 4:**
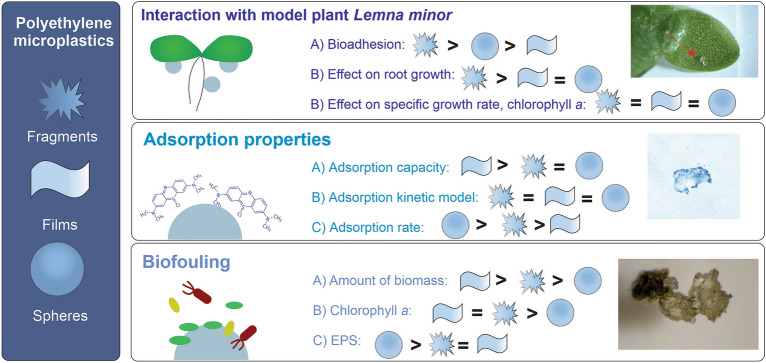
Summarized results of the study.

Overall, it is evident that different shapes of MPs can affect their interactions with their surroundings (Fig. 4). They differently attach to the biotic surfaces, the adsorption of pollutant can proceed in various extent, and also biofouling showed to be affected by MP shape. Fragments adhered to the plant biomass to the highest extent and affected plant growth. The films exhibited the highest adsorption capacity and biofilm growth, while the surface of spherical MPs was not favourable for the biofilm growth especially in case of photosynthetic microorganisms. From the results of this study, it is evident that the shape of MPs significantly affects not only ecotoxicity but also other environmentally relevant processes.

## Material and methods

### Microplastics

PE MPs in the shape of fragments, spheres, and films were used in this study. Fragments were extracted from a facial scrub as described in Kalčíková et al.^31^. Briefly, cosmetic product was dissolved in a warm deionised water and filtered (pore size 4–12 µm, Whatman™, USA). Fragments were washed few times with deionised water and then dried in a laboratory dryer (60 ± 2 °C). Spheres with the size specification 90–106 µm (CPMS-0.96) were purchased from a Cospheric LLC (USA). Films were prepared by grinding a shopping bag in a centrifugal mill (ZM 200, Retsch, Germany). The grinding speed was approximately 15,000 rpm and during the grinding, the blades and shopping bag sheets were cooled with dry ice. The mesh size of 250 µm was used to separate the smaller particles (already ground) from the larger particles.

All three types of MPs were characterized in terms of their chemical composition, morphology, shape, size, number of particles per mass, specific surface area, and crystallinity. Chemical composition was determined by Fourier-transform infrared spectroscopy (FTIR, Spectrum Two FT-IR spectrometer, PerkinElmer, USA) with an UATR module. The wavenumber ranged from 4000 to 450 cm^−1^, with a resolution of 2 cm^−1^ (10 scans). Background and ATR correction of the spectra were applied. Optical microscope Imager.Z2m (Zeiss, Germany) and field-emission scanning electron microscope Ultra Plus (Zeiss, Germany) were used for determination of the shape and surface morphology, respectively. Prior to the FE-SEM analysis, samples were coated with a thin Au/Pd layer. Size of MPs was determined by a laser diffraction analyser (S3500 Bluewave, Microtrac, Germany) using a dry unit. The measurement for each MPs was repeated three times and the results were expressed as the number particle size distribution. The number of particles per mass of MPs was determined as described in Rozman and Kalčíková^45^. Briefly, approximately 1–2 mg of MPs was weighted and counted under a stereo microscope (SMZ-171, Miotic, China). The procedure was repeated ten times. The specific surface area was determined according to the Brunauer, Emmett and Teller (BET) method by an ASAP 2020 instrument (Micromeritics Instrument Corporation, USA). Each type of MPs was degassed for 2 h under a vacuum (6 mmHg) at 50 °C before starting the measurement. N_2_ isotherms were obtained based on the BET specific surface area that was determined at six points in the pressure region (p/p_0_) from 0.01 until 0.30 using the BET equation^46^.

The crystallinity was determined by X-ray powder diffraction method and Rietveld refinement method for quantitative phase analysis. Prior to X-ray analysis, all three MPs were sieved through a sieve with 125 μm mesh size to obtain the finest fraction of samples. Measurements were made by a powder diffractometer (PANalytical X'Pert PRO MPD, Malvern Panalytical, USA) in the range 2*θ* from 10° to 80° in steps of 0.034°, using CuKα X-ray light with a wavelength of 1.5406 Å. The crystalline phases presented in samples were characterized using the Crystallographica Search-Match (CSM) software and standards. Quantitative analysis was performed using the Rietveld refinement method. This approach considers the entire diffractogram of the sample and calculates a separate diffractogram for each phase presented in the sample, considering background, preferred orientation, peak overlap, and other errors to obtain the best possible match with the recorded pattern. Samples were premixed with a known amount of a quantitative analysis standard (corundum) that is assumed to be completely crystalline. A standard for quantitative analysis (corundum) was added to the samples at an approximate weight ratio of 80:20 (sample:standard) and homogenised in a mortar. The exact masses of sample and standard were considered in the final calculation of composition and proportion of amorphous and crystalline phases.

### Interactions with plants

The interactions between MPs and an aquatic plant, duckweed *Lemna minor*, were studied in terms of their ecotoxicity and ability to adhere to the plant biomass. The plant was from a permanent laboratory culture cultivated under constant laboratory conditions (24 ± 1 °C, light/dark photoperiod of 16/8 h). Ecotoxicity tests were performed according to the standard procedure^47^. Briefly, ten uniform fronds with roots removed were placed in a 100 mL glass breaker containing 50 mL Steinberg medium. The concentration of each type of MPs was 10,000 particles/L and each treatment (including the control without MPs) was replicated six times. The concentration was selected based on the results of our previous studies^5,6,22,45^ and because concentrations of several thousand particles/L were detected in natural lakes^48^, therefore, selected concentration can be considered as a ˝hot spot˝ concentration. The exposure time was 168 h in a climate chamber at a constant temperature (24 ± 1 °C), a light intensity of 7000 ± 500 lx, and a light/dark photoperiod of 16/8 h.

After 168 h, specific growth rate, root length, and chlorophyll *a* content were determined. First, the number of fronds was counted and the specific growth rate was calculated according to the standard^47^. Root length of ten randomly selected plants in each replicate was measured using a millimeter paper^22^. Chlorophyll *a* content was determined as described in Kalčíková et al.^49^. Briefly, 15 mg of the fresh plant was homogenized in 2.1 mL cold 95% *v/v* ethanol solution. The homogenate was incubated in a freezer at −18 ± 2 °C for 24 h. The absorbance of the supernatant was measured at 664.2 nm and 648.6 nm (Cary 50 UV–Vis spectrophotometer, Agilent Technologies, USA) and the chlorophyll *a* content was calculated according to Lichtenthaler^50^.

Adhesion of MPs was evaluated as described in Rozman et al.^51^. In each replicate, approximately 30 mg of the plant was collected and washed with deionized water (approximately 2 mL per two fronds). Washing water was then filtered (S-Pak filter, pore size 0.22 µm, Merck Millipore, USA) and particles were counted using a stereo microscope (SMZ-171, Motic, China). These particles were classified as weakly adhered. Strongly adhered particles were recovered by digestion of plant biomass using Fenton oxidation (2 mL of 30% *w/w* H_2_O_2_ and 2 mL of 15 g/L Fe_2_SO_4_ · 7H_2_O with 6 mL/L of H_2_SO_4_ (97% *v/v*)). Digestion was carried out at room temperature (22 ± 2 °C) for 24 h. The digestate was filtered (S-Pak filter, pore size 0.22 µm, Merck Millipore, USA) and the particles were counted under the stereo microscope. The results were expressed as the number of particles per mg fresh weight. The effect of Fenton oxidation on the particles was also evaluated by determining the mass loss during the process. Briefly, 2 mL of both reagents for Fenton oxidation were added to approximately 20 mg of MPs and incubated for 24 h under the same conditions as the digestion of plant tissue was performed. The digestate was filtered (S-Pak filter, pore size 0.22 µm, Merck Millipore, USA) and MPs were weighted again. The procedure was replicated four times for each type of MPs. The effect of Fenton oxidation on the particles was considered negligible, as the weight of fragments, spheres, and films was reduced by 1.06 ± 0.15%, 2.24 ± 2.02%, and 2.36 ± 0.82%, respectively, after Fenton oxidation.

### Adsorption properties

The differences in adsorption of a model compound—MB on different shapes of PE MPs were studied, because it was previously shown that it is well adsorbed on PE^52^. Four replicates were prepared for each shape and four replicates for the blank (without MPs).

Each replicate contained 20 mL of 1 mg/L of the MB (Kemika, Croatia) prepared in a deionized water in a 100 mL Erlenmeyer flask. The concentration of MPs in the adsorption experiment was 10 g/L. It has to be noted that this concentration is higher than those found in the environment, but the main aim of the study was to provide comparison of the adsorption properties of MPs with different shapes and a higher concentration increases the accuracy of the analytical determination. Moreover, this is the concentration used in previous adsorption studies^53,54^. The adsorption experiment proceeded for 30 min at room temperature (22 ± 2 °C) under continuous shaking (160 rpm, orbital shaker Orbit 1900, Labnet, USA). Samples were collected after 0, 1, 3, 5, 8, 10, 15, and 30 min and 200 µL of solution from each replicate was collected at each sampling time. The concentration of MB in the samples was determined at 665 nm with a UV–Vis spectrophotometer (Shimadzu 2600, Japan) by using a calibration curve. The concentration of MB in the blank (without MPs) decreased for 9 ± 1%, and this decreasing was taken into account in the final calculations.

To describe the adsorption process, two kinetic models were applied and compared: the pseudo-first order model (Eq. 1) and the pseudo-second order model (Eq. 2)^55,56^:

1 $${q}_{t}= {q}_{e}(1- {e}^{-{K}_{1}t})$$

2 $${q}_{t}= \frac{{q}_{e}^{2}{K}_{2}t}{1+ {q}_{e}{K}_{2}t}$$

where *K*_1_ is the pseudo-first order rate constant (1/min), *K*_2_ is the pseudo-second order rate constant (g/µg·min), *q*_*e*_ is the amount of MB adsorbed at equilibrium (µg/g), *q*_*t*_ is the amount of MB adsorbed at any time (µg/g), and *t* is the time of adsorption (min).

### Biofouling

MPs were exposed to natural water from a local stream (Glinščica, Ljubljana, Slovenia, 46.051131, 14.467769) for 6 weeks (until biofilm was visible and detectable on all three types of MPs) to evaluate how the shape of MPs affects the development of biofilm. Each type of MPs (150 mg) was transferred to an Erlenmeyer flask and 150 mL of stream water was added resulting in concentration of MPs 1 g/L. The flasks were placed on an orbital shaker (Orbit 1900, Labnet, USA) at 125 rpm. Each week, MPs were filtered (cellulose filter, MN 617, Macherey–Nagel, Germany) by vacuum filtration, and transferred to another Erlenmeyer flask containing 150 mL of fresh stream water. The experiment proceeded under constant temperature (22 ± 1 °C), light intensity of 945 ± 59 lx, and a light/dark photoperiod of 16/8 h. After 6 weeks, MPs were filtered and immediately analysed.

To assess differences in biofilm development, the amount of biofilm, the amount of extracellular polymeric substances (EPS), and chlorophyll *a* contents were determined as described in Rozman et al.^39^. The amount of biofilm was determined by weighing the aged MPs before and after biofilm digestion by Fenton oxidation (described in “Interactions with plants”). The amount of biofilm was determined in three replicates for each type of MPs, and approximately 30 mg of MPs with biofilm was digested for each replicate. Results were expressed as a percentage of the total mass of aged MPs.

The EPS content within the developed biofilm was determined according the method of Dubois et al.^57^ with some minor modifications. Briefly, approximately 15 mg of MPs with biofilm (in three replicates for each type of MPs) were added to the 3 mL of deionised water and incubated at 80 ± 1 °C for 30 min. After cooling to room temperature, the particles were removed (cellulose acetate filters with a pore size of 0.45 µm, Merck Millipore, USA) and 1 mL of the filtrate was added to 0.5 mL of 6% *w/v* phenol and 2.5 mL of H_2_SO_4_ (97% *v/v*). The absorbance was measured at 490 nm (Cary 50 UV–Vis spectrophotometer, Agilent Technologies, USA) after cooling to room temperature. The calibration curve was prepared using D-( +)-glucose.

Chlorophyll *a* content was determined as described in Kalčíková et al.^49^. Briefly, 15 mg of MPs with biofilm (in three replicates for each type of MPs) were used for the analysis, homogenized in 1.8 mL cold 95% *v/v* ethanol solution, and then the procedure was performed as described in “Interactions with plants”*.*

### Statistical analysis

The results are expressed as arithmetic mean ± standard deviation. The normality and homogeneity of variances were tested with the Shapiro–Wilk test and Levene’s test, respectively. Because the data were normally distributed and homogeneous, one-way ANOVA statistical test was applied (Supplementary information, Table S1), followed by the Tukey test. Differences were considered statistically significant if *p* < 0.05. All analyses were performed using OriginPro 2022b software (OriginLab Corp., USA).

### Supplementary Information

Supplementary Table S1.

## Data Availability

The data presented in this study are available on request from the corresponding author.
