# Supplementary material for: Insights into the shape-dependent effects of polyethylene microplastics on interactions with organisms, environmental aging, and adsorption properties
Source: Sci Rep. 2023 Dec 13;13:22147. doi: 10.1038/s41598-023-49175-1 (PMC10719240; doi:10.1038/s41598-023-49175-1)
Supplement: Supplementary file 1 — Supplementary Table S1. [file 41598_2023_49175_MOESM1_ESM.pdf]

Supplementary information

Insights into the shape-dependent effects of polyethylene microplastics on interactions with organisms, environmental aging, and adsorption properties

Ula Rozman, Barbara Klun, Aleksandra Kuljanin, Tina Skalar, Gabriela Kalčíková*

University of Ljubljana, Faculty of Chemistry and Chemical Technology, 113 Večna pot, SI-1000 Ljubljana, Slovenia

*gabriela.kalcikova@fkkt.uni-lj.si

Table S1

Table S1. *F* values for ANOVA statistical testing.

		F (/)
Interactions with plants	Specific growth rate	1.33
	Root length	4.75
	Chlorophyll *a*	1.50
Adsorption properties		76.26
Biofouling	The amount of biofilm	15.25
	EPS	16.33
	Chlorophyll *a*	20.30
